# Robotic-Assisted Total Pelvic Exenteration for Rectal Cancer Using the Hugo™ RAS System: First Case Report

**DOI:** 10.3390/jcm14186603

**Published:** 2025-09-19

**Authors:** Kosuke Hiramatsu, Shigeo Toda, Shuichiro Matoba, Daisuke Tomita, Yusuke Maeda, Naoto Okazaki, Yudai Fukui, Yutaka Hanaoka, Masashi Ueno, Suguru Oka, Tomoaki Eguchi, Hiroya Kuroyanagi

**Affiliations:** 1 Colorectal Surgery Division, Department of Gastroenterological Surgery, Toranomon Hospital, 2-2-2 Toranomon, Minato-ku, Tokyo 105-8470, Japan; hiramatsu.v05@toranomon.gr.jp (K.H.); shigeo-toda@toranomon.gr.jp (S.T.);; 2 Division of Colorectal Surgery, Department of General Gastroenterological Surgery, Toho University Omori Medical Center, 6-11-1 Omori-Nishi, Ota-ku, Tokyo 143-8541, Japan; 3 Department of Urology, Toranomon Hospital, 2-2-2 Toranomon, Minato-ku, Tokyo 105-8470, Japan; 4 Department of Plastic Surgery, Toranomon Hospital, 2-2-2 Toranomon, Minato-ku, Tokyo 105-8470, Japan

**Keywords:** robotic surgery, total pelvic exenteration, Hugo™ RAS system, locally advanced rectal cancer, minimally invasive approach, intracorporeal urinary diversion, prostatic invasion, pelvic reconstruction

## Abstract

**Introduction**: Total pelvic exenteration (TPE) is a radical procedure for advanced pelvic malignancies involving adjacent organs. The Hugo™ RAS System is a novel robotic platform, but its application in TPE has not previously been reported. We describe the first case of robotic-assisted TPE using Hugo™ RAS in a patient with locally advanced rectal cancer invading the prostate. **Methods**: A 69-year-old male with mucous and bloody stools was diagnosed with cT4b (prostate, levator ani muscle) N0M0 rectal cancer. After short-course radiotherapy (25 Gy/5 fractions), robotic-assisted TPE was performed. Port placement was planned to coincide with future colostomy and urostomy sites to minimize abdominal wall trauma. En bloc resection was achieved, followed by pelvic reconstruction with a gluteus maximus musculocutaneous flap and fascia lata autograft. Urinary diversion was completed with a robotic intracorporeal Wallace-type ileal conduit. **Results**: The operation lasted 17 h 56 min, with 175 mL blood loss. Postoperatively, Clavien–Dindo grade IIIa paralytic ileus occurred but was managed conservatively. Pathology revealed pT4b (prostate) N1a M0 disease with negative circumferential margin (11 mm). No recurrence was observed at 9 months. **Conclusions**: This case highlights the technical feasibility and safety of Hugo™ RAS-assisted TPE. Further clinical experience is needed to confirm reproducibility and oncologic safety.

## 1. Introduction

Robotic-assisted surgery has emerged as a promising minimally invasive approach for improving surgical precision and patient outcomes in the treatment of colorectal cancer. The advantages of robotic-assisted surgery, including multi-jointed articulation and high-resolution three-dimensional imaging, are particularly beneficial in rectal surgery, which requires precise manipulation within the narrow pelvic cavity. These features have been shown to contribute to favorable short-term surgical and oncological outcomes [1,2,3].

The Hugo™ RAS system (Medtronic, Minneapolis, MN, USA) represents a highly anticipated alternative to the da Vinci^®^ Surgical System (Intuitive Surgical, Sunnyvale, CA, USA), having received European certification for use in gynecologic and urologic surgery in early 2022 and for general surgery later the same year. The Hugo™ RAS system was approved by the Japanese Ministry of Health, Labor and Welfare for gastroenterological surgery in May 2023. Toyota et al. reported the first case of the rectal cancer surgery using the Hugo™ RAS system in Japan [4]. Since February 2024, we have performed colorectal cancer surgeries using the Hugo™ RAS system. To the best of our knowledge, this is the first reported case of total pelvic exenteration (TPE) using the Hugo™ RAS system for locally advanced rectal cancer (LARC) with prostatic invasion, and we present it here.

## 2. Materials and Methods

### 2.1. Patient

A 69-year-old male presented to a local clinic with a chief complaint of mucous and bloody stools persisting for one month. Two months earlier, his food intake had decreased due to mild postprandial abdominal pain, and he had lost 2 kg over the preceding three months. Colonoscopy revealed a circumferential tumor in the rectum, raising suspicion for rectal cancer. He was subsequently referred to our hospital for further evaluation and management.

He had a medical history of hypertension and dyslipidemia, for which he was receiving ongoing medication. He had undergone surgery for a left vestibular schwannoma ten years earlier, resulting in complete left-sided hearing loss. His family history was notable for gastric cancer in his father.

Laboratory investigations revealed mild anemia with a hemoglobin level of 12.5 g/dL. Tumor markers were elevated: carcinoembryonic antigen (CEA), 48.8 ng/mL; and carbohydrate antigen 19-9 (CA19-9), 860 U/mL. Colonoscopy showed a circumferential, ulcerated, infiltrative lesion with limited mobility, extending from 3 cm to 10 cm from the anal verge. Biopsy revealed a well to moderately differentiated tubular adenocarcinoma. The endoscope could pass through the lesion with difficulty.

**Figure 1:**
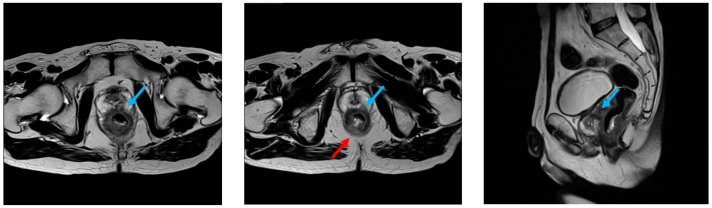
Preoperative T2-weighted MRI revealed a circumferential tumor in the lower rectum, with suspected invasion into the prostate (blue arrow) and levator ani muscle (red arrow).

For staging purposes, contrast-enhanced computed tomography (CT) and magnetic resonance imaging (MRI) were performed. Although no distant metastasis or regional/lateral lymph node involvement were detected, the tumor appeared to extensively invade the prostate and the levator ani muscle (Figure 1). Based on these findings, the clinical stage was determined to be cT4b (prostate, levator ani muscle), N0, M0, corresponding to stage IIc.

The tumor exhibited extensive invasion of the prostate and anal sphincter. We concluded that, even if tumor shrinkage were achieved through neoadjuvant therapy, TPE would still be required. Therefore, neoadjuvant short-course radiotherapy with a total dose of 25.0 Gy in 5 fractions was administered for local control followed by robotic-assisted TPE.

### 2.2. Surgical Setup

**Figure 2:**
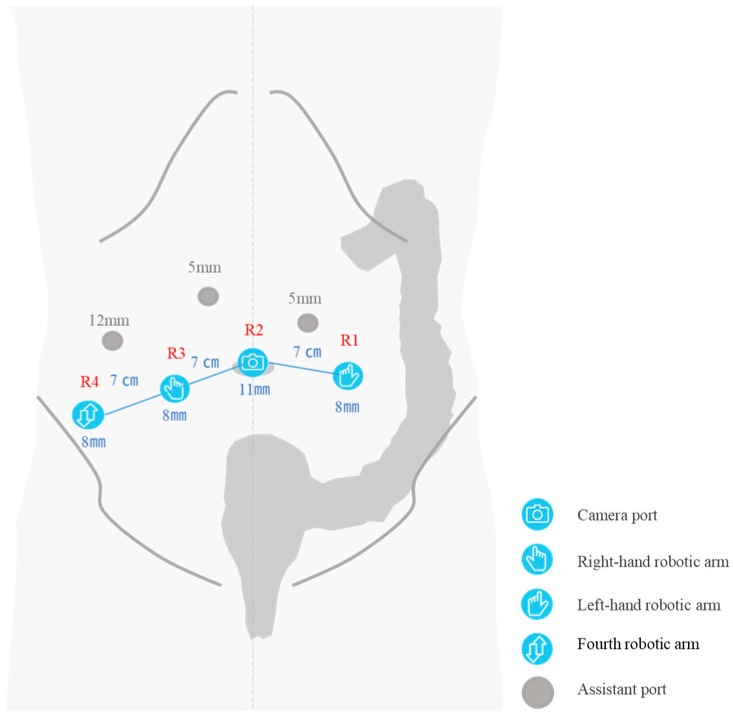
Port placement for robot-assisted TPE using the Hugo™ RAS system: R1 and R3 ports were placed at the planned sites for colostomy and ileal conduit construction, respectively. The left-sided assistant port was used by the urology team.

The patient was placed in the lithotomy position with a 15-degree Trendelenburg tilt. The robotic ports were inserted as shown in Figure 2. The first port (R2), an 11 mm camera port, was inserted at the umbilicus using the open technique. The R1 port was inserted at the site designated for colostomy creation, and the R3 port was inserted at the site designated for ileal conduit construction. A 12 mm assistant port with an AirSeal^®^ trocar was employed. A polyvinyl chloride (PVC) drainage tube with a 2 mm outer diameter was inserted separately through the right abdominal wall during surgery for exudate drainage.

**Figure 3:**
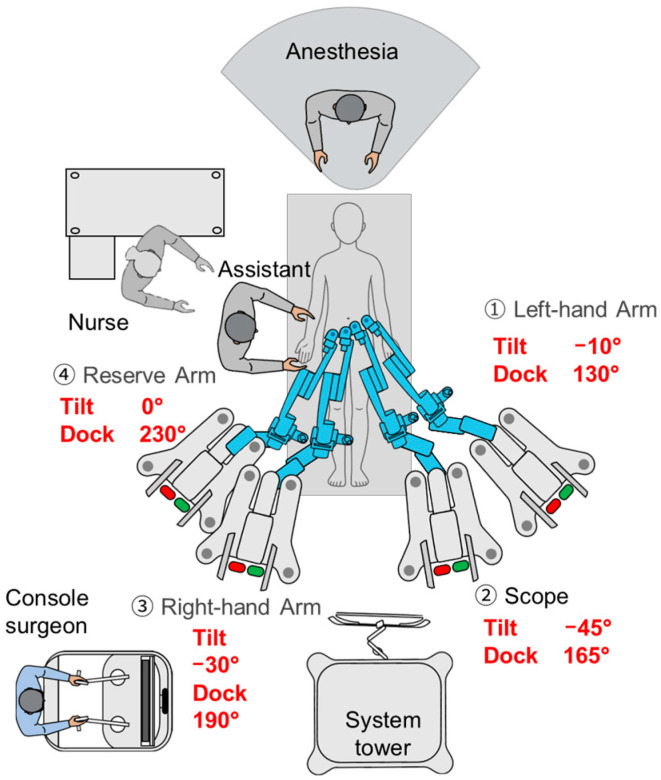
Operating room layout and arm cart configuration.

The Hugo™ RAS system was utilized. Arm carts 1 and 2 were positioned on the patient’s left side, and arm carts 3 and 4 on the patient’s right side, followed by docking with the robotic trocars. Tilt and docking angles were set at −0° and 130° for arm 1, −45° and 155° for arm 2, −30° and 190° for arm 3 and −0° and 225° for arm 4, respectively (Figure 3). The instruments used were a bipolar fenestrated grasper for arm 1, a 30° oblique-viewing laparoscope for arm 2, monopolar-curved shears for arm 3 and double fenestrated grasper for arm 4.

### 2.3. Surgical Procedure

**Figure 4:**
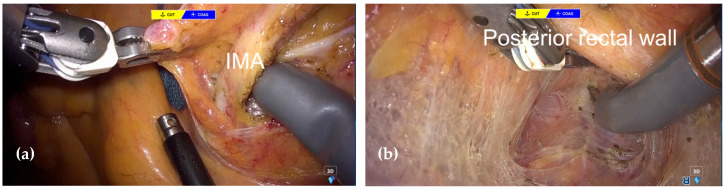
Intraoperative finding during robotic TPE. (**a**) proximal lymphadenectomy. (**b**) posterior dissection of the rectum.

After port insertion and intraperitoneal exploration to rule out peritoneal and distant metastasis, the robotic-assisted TPE was performed. A peritoneal incision was initiated on the right side at the level of the sacral promontory, and dissection was carried into the retrorectal space. Dissection was then continued cranially to mobilize the sigmoid mesocolon and identify the inferior mesenteric artery (IMA), which was divided at its root (Figure 4a). The inferior mesenteric vein and the left colic artery were also divided at the same level. Both ureters were identified and taped, followed by mobilization toward the bladder. Dissection was carried out along the presacral fascia on the dorsal side of the rectum, reaching the supralevator space (Figure 4b).

**Figure 5:**
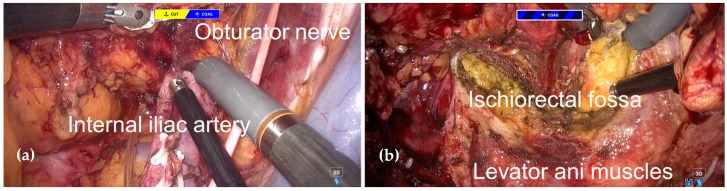
Intraoperative finding during robotic TPE. (**a**) Right lateral lymphadenectomy. (**b**) Division of the levator ani muscles.

Bilateral lateral lymph node dissection was then performed. On both sides, the main trunk of the internal iliac artery (IIA) and the obturator nerve (ON) were preserved (Figure 5a). The umbilical arteries, superior vesical vessels, obturator vessels, and inferior vesical vessels were divided. Lymphadenectomy was performed for the internal iliac proximal (#263p), internal iliac distal (#263d), and obturator (#283) lymph nodes, in accordance with the Japanese Classification of Colorectal, Appendiceal, and Anal Carcinoma [5], and the levator ani muscle was exposed caudally.

Dissection was extended by transecting the pelvic plexus to connect the lateral and posterior rectal planes. The levator ani muscles were then divided bilaterally and posteriorly, allowing entry into the fat plane of the ischiorectal fossa (Figure 5b). Subsequently, both ureters were transected distally near the bladder, and the sigmoid colon was transected using the SIGNIA™ stapling system (Medtronic, Minneapolis, MN, USA).

**Figure 6:**
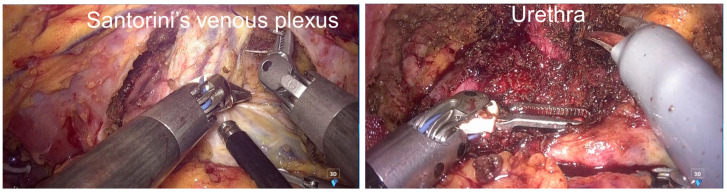
Intraoperative finding during robotic TPE. Santorini’s venous plexus was ligated, and the urethra was transected.

The procedure then proceeded anteriorly. The distal portion of the umbilical artery was divided, and the bladder was mobilized from the anterior abdominal wall. The space of Retzius was developed and connected with the bilateral paravesical spaces. Santorini’s venous plexus was ligated using 3-0 V-loc™ (Medtronic, Minneapolis, MN, USA) (Figure 6). The deep dorsal vein of the penis was divided and sealed with a LigaSure™ Maryland jaw device (Medtronic, Minneapolis, MN, USA). Finally, the urethra was transected using the SIGNIA™ stapling system.

The robotic phase of the procedure was temporarily concluded, and the operation proceeded to the perineal phase. To prevent tumor spillage, the anus was closed with a purse-string suture. A perineal skin incision was then made, and dissection was carried out to connect with the abdominal dissection plane, allowing en bloc resection.

Next, the patient was repositioned to the jackknife position, and perineal reconstruction was performed by the plastic surgeons using a gluteus maximus musculocutaneous flap and a fascial graft harvested from the fascia lata. A fascia lata autograft was secured to the pelvic floor to obliterate dead space and reinforce the cavity, followed by advancement of a unilateral gluteus maximus flap [6].

**Figure 7:**
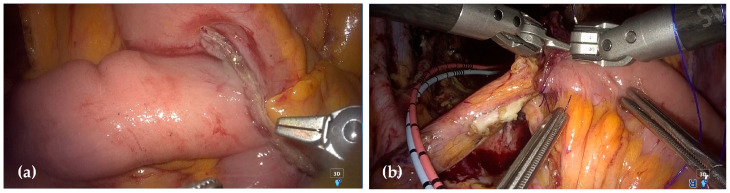
Intraoperative finding during robotic TPE. (**a**) Intracorporeal ileo-ileal anastomosis using the overlap method. (**b**) Robotic ileal conduit urinary diversion with Wallace-type uretero-ileal anastomosis.

The Hugo™ system was re-docked using the same configuration as before. At this time, an additional 5 mm laparoscopic assist port was placed on the patient’s left side, positioning the assistant in accordance with the standard setup used in urological robotic surgery. First, an ileal conduit was constructed using a 20–40 cm segment from the distal ileum. Intracorporeal ileo-ileal anastomosis was performed using the overlap method with the SIGNIA™ stapling system (Figure 7a). A robotic ileal conduit urinary diversion was constructed by the urologists, employing a Wallace-type uretero-ileal anastomosis (Figure 7b). After wound closure, a colostomy was created at the R1 port site using the sigmoid colon, and a urostomy was constructed at the R3 port site.

## 3. Results

The total operative time and anesthesia time were 17 h and 56 min and 19 h and 2 min, respectively. The breakdown was as follows: 7 h and 8 min of console time until specimen removal, 1 h and 31 min for perineal reconstruction, and 3 h and 43 min of console time for urinary tract reconstruction. Estimated blood loss was 175 mL, and no intraoperative complications occurred. The patient spent one day in the intensive care unit. Oral intake was initiated on postoperative day 3.

The patient developed postoperative paralytic ileus, which required placement of a decompression tube for six days. This complication was classified as Clavien-Dindo grade IIIa. The total postoperative stay was 34 days.

**Figure 8:**
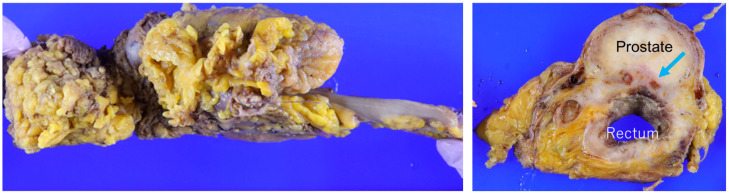
En bloc surgical specimen obtained by robotic-assisted TPE. The blue arrow indicates rectal cancer invasion into the prostate.

Pathological examination revealed T4b (prostate) N1a M0, with one of 37lymph nodes positive. No lateral lymph node metastasis was identified. The circumferential resection margin (CRM) was negative, with a clearance of 11 mm from the tumor (Figure 8). At the 9-month follow-up, no evidence of recurrence was observed.

## 4. Discussion

This report presents, to our knowledge, the first case of TPE performed using the Hugo™ RAS system for LARC. The procedure was completed safely, and pathological examination confirmed an R0 resection. As this was the first TPE performed using the Hugo™ RAS system, the operative time was slightly longer than usual. However, the procedure was completed without intraoperative complications or technical issues. Although the patient experienced a minor postoperative complication, overall recovery was favorable. Despite being limited to a single case, this report suggests that the Hugo™ RAS system may be a feasible option for pelvic cancer surgeries requiring adjacent organ resection.

TPE was initially performed with palliative intent in patients with recurrent cervical cancer in 1948 [7]. Laparoscopic TPE was first reported by Pomel et al. in 2003 in a patient with recurrent cervical cancer [8]. This landmark case demonstrated the technical feasibility of performing TPE via a minimally invasive approach. The first report of robotic TPE for a patient with gynecologic malignancy was published by Lim in 2009 [9]. Following this, additional cases of robotic TPE for advanced pelvic malignancies have been reported [10,11,12,13], indicating increasing acceptance of robotic systems in the management of advanced pelvic malignancies. A recent multicenter propensity-matched analysis from Japan compared robotic, laparoscopic, and open pelvic exenteration for pelvic malignancies [14]. Robotic exenteration was associated with significantly reduced intraoperative blood loss and shorter hospital stays. Importantly, these benefits were achieved without compromising oncologic outcomes.

This procedure was designed with an emphasis on minimizing abdominal wall trauma through strategic port placement and technical modifications. Although arm positioning and system setup followed the standard protocol used at our institution for rectal cancer surgery with the Hugo™ RAS system, the port placement was modified to correspond with the planned sites for the colostomy and urostomy creation. The R1 port is typically inserted in the patient’s left upper abdomen, with the four ports aligned in an oblique, ascending straight line toward the left upper quadrant. Lowering the R1 port caudally may slightly limit the mobility of the R1 arm during proximal dissection around IMA; however, this does not cause significant difficulty during pelvic procedures. Therefore, the benefit of reducing abdominal wall incisions is considered substantial.

In addition, the intracorporeal ileo-ileal anastomosis using the overlap method, ileal conduit construction, Wallace-type uretero-ileal anastomosis were all performed under robotic assistance. Although these intracorporeal techniques may have contributed to a longer operative time, they likely helped minimize abdominal wall trauma.

In conclusion, we safely performed robotic-assisted TPE using the Hugo™ RAS system in a patient with LARC. This case demonstrates the technical feasibility of the Hugo™ RAS system for complex pelvic procedures. However, as this is a single case report, further accumulation of cases and long-term follow-up are required to validate the safety, efficacy, and reproducibility of this approach.

## Data Availability

The original contributions presented in the study are included in the article, further inquiries can be directed to the corresponding author.
